# Permitted speed decision of single-unit trucks with emergency braking maneuver on horizontal curves under rainy weather

**DOI:** 10.1371/journal.pone.0261975

**Published:** 2021-12-30

**Authors:** Menghua Yan, Jinliang Xu, Shuo Han, Tian Xin, Ouyu Wang, Zemin Yi, Zhaoxin Liu

**Affiliations:** 1 School of Highway, Chang’an University, Xi’an, China; 2 College of Transportation Engineering, Chang’an University, Xi’an, China; 3 Shandong Hi-speed Infrastructure Construction Co., Ltd., Jinan, China; Tongji University, CHINA

## Abstract

Under adverse weather conditions, visibility and the available pavement friction are reduced. The improper selection of speed on curved road sections leads to an unreasonable distribution of longitudinal and lateral friction, which is likely to cause rear-end collisions and lateral instability accidents. This study considers the combined braking and turning maneuvers to obtain the permitted vehicle speed under rainy conditions. First, a braking distance computation model was established by simplifying the relationship curve between brake pedal force, vehicle braking deceleration, and braking time. Different from the visibility commonly used in the meteorological field, this paper defines "driver’s sight distance based on real road scenarios" as a threshold to measure the longitudinal safety of the vehicle. Furthermore, the lateral friction and rollover margin is defined to characterize the vehicle’s lateral stability. The corresponding relationship between rainfall intensity-water film thickness-road friction is established to better predict the safe speed based on the information issued by the weather station. It should be noted that since the road friction factor of the wet pavement not only determined the safe vehicle speed but also be determined by the vehicle speed, so we adopt Ferrari’s method to solve the quartic equation about permitted vehicle speed. Finally, the braking and turning maneuvers are considered comprehensively based on the principle of friction ellipse. The results of the TruckSim simulation show that for a single-unit truck, running at the computed permitted speed, both lateral and longitudinal stability meet the requirements. The proposed permitted vehicle speed model on horizontal curves can provide driving guidance for drivers on curves under rainy weather or as a decision-making basis for road managers.

## 1. Introduction

The design criteria [1] and most of the current advisory speed models [2, 3] on horizontal curves are for safety and comfort matters under normal weather conditions. They are for steady-state driving vehicles without adequate consideration of the situations when vehicles may have to apply emergency braking which reduces the tire/pavement friction available to the lateral direction to maintain turning path on horizontal curves. Two main situations require emergency braking collision avoidance maneuver: a) an object suddenly falls onto the lane, an animal suddenly crossing the road, or the front vehicle suddenly stops due to a malfunction within the demanded stopping sight distance, b) the sight distance is less than the demanded stopping sight distance caused by adverse weather or dim light. These scenarios occur rarely but are unavoidable, especially dangerous for curved road sections, where part of the tire/pavement friction has been distributed in the lateral direction to maintain a turning path. Therefore, management measures (such as speed guidance) should be taken to ensure the safety of this scenario for the built roads. Research shows that human error accounts for 94% of the final failure in the causal chain of crash events [4]. An analysis of the NMVCCS database shows that planning and decision errors (39%) are the most common contributing factors of driver-related crashes. Further, speed (23%) is the most common planning and deciding factor [5]. In addition, the weather is also an important factor to be considered, because the low-visibility and low friction caused by adverse weather will exacerbate the impact of road alignment. Roughly 24% of 6,301,000 vehicle crashes are identified as taking place in adverse weather (i.e., rain/snow/sand/fog) or even on slick pavement (i.e., wet pavement, snowy/slushy pavement, or icy pavement). On average, there are approximately 7,000 fatalities and over 629,000 injuries in weather-related crashes annually [6]. Thus, it is important to determine a proper safe speed for individual vehicles, especially for built roads whose geometric alignment is unlikely to be modified and affect the safe vehicle speed.

Many studies have shown that the average vehicle crash rate on curved road sections is considerably higher than that on straight road sections [7, 8]. The vehicle lateral stability on horizontal curves has been studied extensively based on lateral acceleration [9–12], roll angle threshold values [13–15], Lateral-Load Transfer Ratio [16, 17], and lateral friction margin [18, 19]. Some studies have conducted preliminary explorations on curve safe speeds. One project has determined the feasibility of in-vehicle dynamic curve-speed warnings to avoid or reduce lane-departure crashes [20]. Deng Zejian et al. established a curve safe speed model introducing a driver behavior influence factor associated with a driving style based on the 28-item Chinese version of the Driver Behaviour Questionnaire [21]. J. Peng et al. present a finite element simulation model to determine the safe vehicle speeds on wet horizontal pavement curves considering the skid resistance requirements [22]. Concerning the vehicle system itself, models with different degrees of freedom were developed to study the dynamic behavior of tractor-semitrailers during different maneuvers. Ellis adopted a dynamic model with 6 DOF to study the effect of the tires on roll motion of tractor-semitrailer through a combined stiffness of individual suspensions and tires [23]. Chen and Tomizuka developed a dynamic model with 5 DOF for lateral control of tractor-semitrailers by neglecting the tractor pitch and bouncing motions [24]. Hyun adopted a 14 DOF model to active rollover control of rollover [25].

Rain is one of the most common bad weather, which has the greatest impact on the number of traffic accidents [26]. Rain is an important weather-related factor leading to casualties [27]. The survey results of FHWA show that 75% of weather-related vehicle crashes happen on wet pavement and 47% occur during rainfall [28]. A review indicates that rain increased the accident rate by 71% and the casualty rate by 49% [29]. Ten-year averages of NHTSA data analyzed by Booz Allen Hamilton indicate that most weather-related crashes take place on wet pavement while rainfall: 73% on wet pavement and 46% during rainfall [6]. Rainy weather conditions often diminish visibility distance and reduce tire/pavement friction. Most of the earlier studies that studied weather impact on traffic safety indicated that crash rates increase during adverse weather as pavement became wet [30, 31]. There are also many studies focused on the impact of reduced visibility caused by adverse weather on traffic flow characteristics, traffic capacity, traffic demand, traffic safety, drivers’ response [32, 33]. Also, many pieces of research directly give friction coefficient based on pavement conditions [34, 35].

In this paper, we first present in Section 2.1 the demand of vehicle longitudinal safety by considering the relationship between the braking distance and the rainy-day sight distance based on real-road scenarios. Section 2.2 presents the demand of vehicle lateral safety based on the equation of motion, lateral friction, and rollover margins. In Section 2.3, the braking and cornering maneuvers are combined through the distribution of longitudinal and lateral friction based on the friction ellipse. Section 2.4 presents the process of determining the permitted vehicle speed based on the analysis in Sections 2.1 to 2.3. In Section 3, the proposed model is validated using TruckSim simulation experiments.

## 2. Methods

The maximum safe speed for curve negotiation depends on the characteristics of the road, vehicle, driver maneuver, and weather. The radius of curvature, slope, and banking are fixed road geometric factors, while the maximum lateral friction factor is the road-dependent factor that varies with the temperature of the road surface, precipitation on the road surface, the tires, and speed of the vehicle. Different types of vehicles have different lateral stability performances on curved road sections. As an important participant in the driving task, the driver’s emergency braking operation will lead to an increase of the longitudinal friction demand which may result in insufficient lateral friction on horizontal curves. The adverse weather may cause the deceleration of the maximum available friction and the driver’s sight distance. The safe speed model could be modified by taking into account these factors.

### 2.1 Longitudinal braking margin

The longitudinal braking margin is defined as the difference between the braking distance and the supplied longitudinal distance (see in Eq 2). A value of zero for the longitudinal braking margin indicates an occurrence of the rear-end collision. The definition of supplied longitudinal distance studied in this paper is similar to the definition of stopping sight distance: sight distance is the length of the roadway ahead that is visible to the driver [1]. The difference is that the available sight distance in AASHTO refers to a distance long enough to enable a vehicle traveling at or near the design speed to stop before reaching a stationary object in its path. It is the sum of the distance traversed during the brake reaction distance and braking distance. A brake reaction time of 2.5 s is adopted in AASHTO. The approximate braking distance of a highway on a grade is as follows:

$$d_{B}=\frac{V^{2}}{254\left[\left(\frac{a}{9.81}\right)\pm G\right]}$$ (1)

*d*_*B*_ = braking distance on grade [m]

*V* = design speed [km/h]

*a* = deceleration [m/s^2^]

*G* = grade, rise/run [m/m]

It can be seen that the AASHTO model for stopping sight distance considers design speed, perception-reaction time, deceleration rate (during braking), and vertical grade.

However, the situation studied in this paper is that the available sight distance caused by bad weather, falling objects, crossing animals, or stopped vehicles is less than the demanded stopping sight distance. Therefore, it is necessary to determine a suitable speed less than the design speed to ensure that the braking distance is shorter than the supplied longitudinal distance.

BM=s−s′ (2)

BM = Longitudinal braking margin

s = Braking distance [m]

s’ = Supplied longitudinal distance [m]

#### 2.1.1 The mass-point model of the braking distance

**Fig 1:**
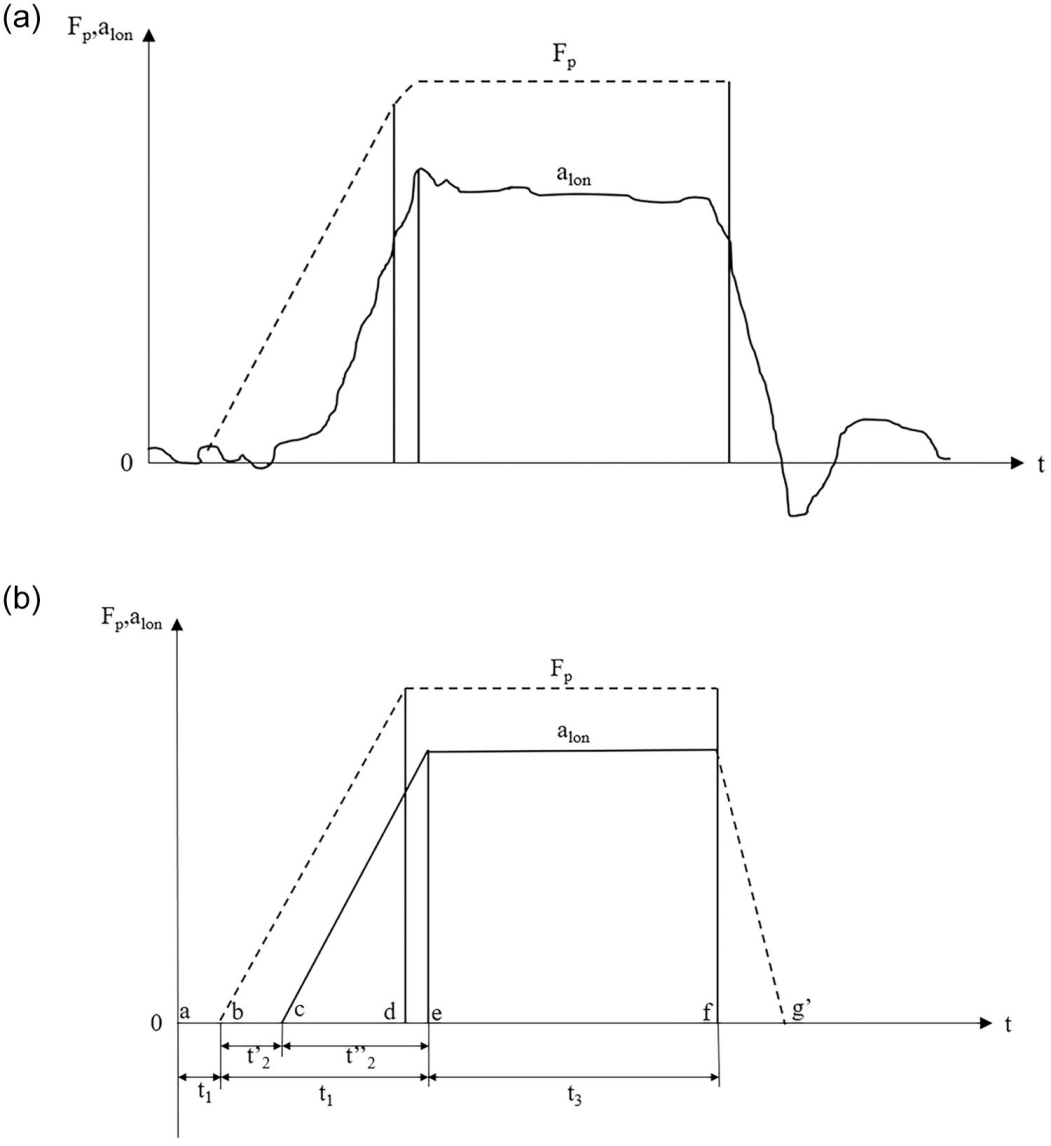
The relationship curve between brake pedal force, vehicle braking deceleration, and braking time: (a) the actual measurement curve, (b) the simplified curve.

This section only considers whether the braking distance of the vehicle meets the safety requirements. In order to simplify the analysis, the mass point model is used to determine the longitudinal braking distance. Fig 1 shows the relationship curve between brake pedal force, vehicle braking deceleration, and braking time. It can be seen from Fig 1(a) that the shape of the instantaneous deceleration curve is too complicated to be represented with the value of a certain point. Therefore, in this paper, the average effect of deceleration in Fig 1(b) is used to approximately reflect the actual deceleration effect in the effective braking stage to simplify the computation. The deceleration is linearly increasing with time during "brake applying stage" until point e where after this point it is considered constant. F_p_ is the brake pedal force, N; a_lon_ is the longitudinal braking acceleration, m/s^2^; t is time, s; a, b, c, d, e, f, and g’ are the points on the t-axis corresponding to the changes of the brake pedal force or the longitudinal braking deceleration. t_1_ is the perception-reaction time, s; t_2_ is the brake reaction and applying time, s; t_2_’ is the brake prepare time, s; t_2_” is the brake applying time, s; t_3_ is the effective braking time, s.

To compute the braking distance, it is necessary to understand the braking process first. It can be seen from Fig 1(b) that the whole process of braking mainly includes five stages: ① the perception stage (a~ b), during which the vehicle travels at a constant speed; ② the brake reaction stage (b~ c), during which the vehicle travels at a constant speed; ③ the brake applying stage (c~ e), during which the braking deceleration increases linearly; ④ the effective braking stage (e~ f), during which the vehicle uses a_lon-max_ to make a uniform deceleration movement with a final speed of 0; ⑤ the brake release stage (f~ g), which is not discussed in this paper as a safe margin. The mass-point model of braking distance proposed by Zhisheng Yu [36] is modified by longitudinal grade [1] and the brake pedal pressure and ABS-related parameter γ [37] as follows:

$$s=\frac{1}{3.6}\left(t_{1}+t_{2}^{\prime }+\frac{t_{2}^{\prime \prime }}{2}\right)u+\frac{u^{2}}{254\left(\frac{\gamma a_{lon-\text{max}}}{g}\pm i\right)}$$ (3)

u = Initial speed of the vehicle when the driver realizes the emergency [km/h]

γ = The brake pedal pressure and ABS-related parameter. γ∈[0, 1] is a parameter linked to the driver’s pressure on the brake pedal and the presence of an ABS in the vehicle. γ = 0.9 is used when the car has an ABS, and γ = 0.7, otherwise [37].

a_lon-max_ = Maximum longitudinal deceleration [m/s^2^]. For road pavement to be able to provide sufficient friction, a value of 4.5 m/s^2^ is adopted as the maximum deceleration in emergency braking operation [38]. In the case where the road pavement cannot provide sufficient friction, the deceleration needs to be determined based on the remaining road friction force after which is distributed to the lateral direction to maintain lateral stability.

g = Gravity [m/s^2^]

i = The percent of grade, rise/ run, m/ m. Positive for upgrades and negative for downgrades. The stopping distances required on upgrades are shorter than that on level roadways; those on downgrades are longer.

Note that the computation of braking distance in Eq (3) does not explicitly consider a truck operation. It is not common in highway engineering to distinguish stopping sight distances between trucks and passenger cars [1]. Studies documented in the literature [39, 40] show that a 2.5-s perception-reaction time regarded as the recommended criterion for stopping sight situations encompasses the capabilities of most drivers (90%), including those of older drivers. The brake reaction and applying time are closely related to the structure of the brake system. The analysis of Zhisheng Yu indicates that when the driver quickly steps on the brake pedal, the hydraulic brake system can operate as short as 0.1s or less; the vacuum booster brake system and the pneumatic brake system are 0.3~0.9s; when the truck has a trailer, the brake working time is sometimes as long as 2s, but the well-designed brake system of automobile and train can be shortened to 0.4s [36]. t_2_ is generally between 0.2 and 0.9 s.

#### 2.1.2 Rainy day sight distance experiment based on real-road scenarios stopping sight distance targets

Rainy weather may cause “splash” and “spray” phenomena, which adversely affect driver sight distance on the wet highway [41]. Noted that the visibility in previous studies about road traffic safety [42–44] is a concept in meteorology, which refers to the greatest distance that a person with normal vision can recognize the target (black, moderate size) from the sky background. As a belt-shaped structure in a three-dimensional space, the background of the target on the road may be mountains, plants, buildings, road surface, etc., not just the sky. The brightness of these backgrounds is lower than that of the sky, which may make the identification of the target more difficult.

**Fig 2:**
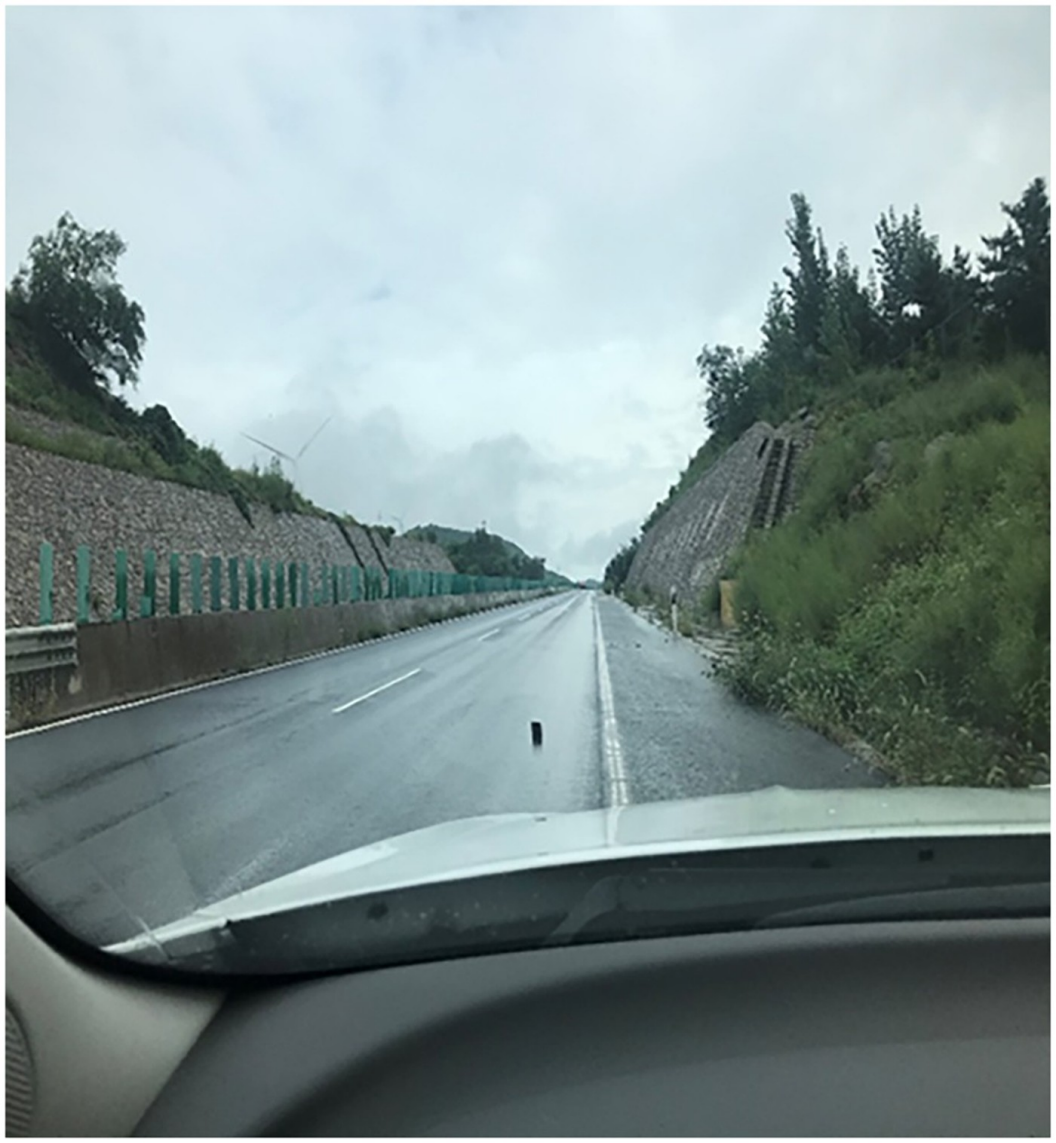
The road experimental scene.

A series of field experiments measuring the sight distance based on real scenarios and stopping sight distance targets under 6 rainfall intensities were carried out from 9 am to 12 am with a wind scale of less than 5.4m/s and without obvious fog (Fig 2). The sight distance based on real scenarios and stopping sight distance targets here refers to the average distance at which the driver can observe the outline of the black cube placed on the road with a side length of ten centimeters. The cube size is determined according to the definition of the stopping distance in the Chinese road regulations [45]. The cube color is determined according to the definition of visibility. The experiment requires two vehicles, one is called the front vehicle and the other is called the rear vehicle. The tester in the front vehicle is responsible for measuring rainfall intensity and placing the target. The tester sits in the co-pilot position of the rear vehicle. The observer in the rear vehicle is responsible for the record of the sight distance.

**Fig 3:**
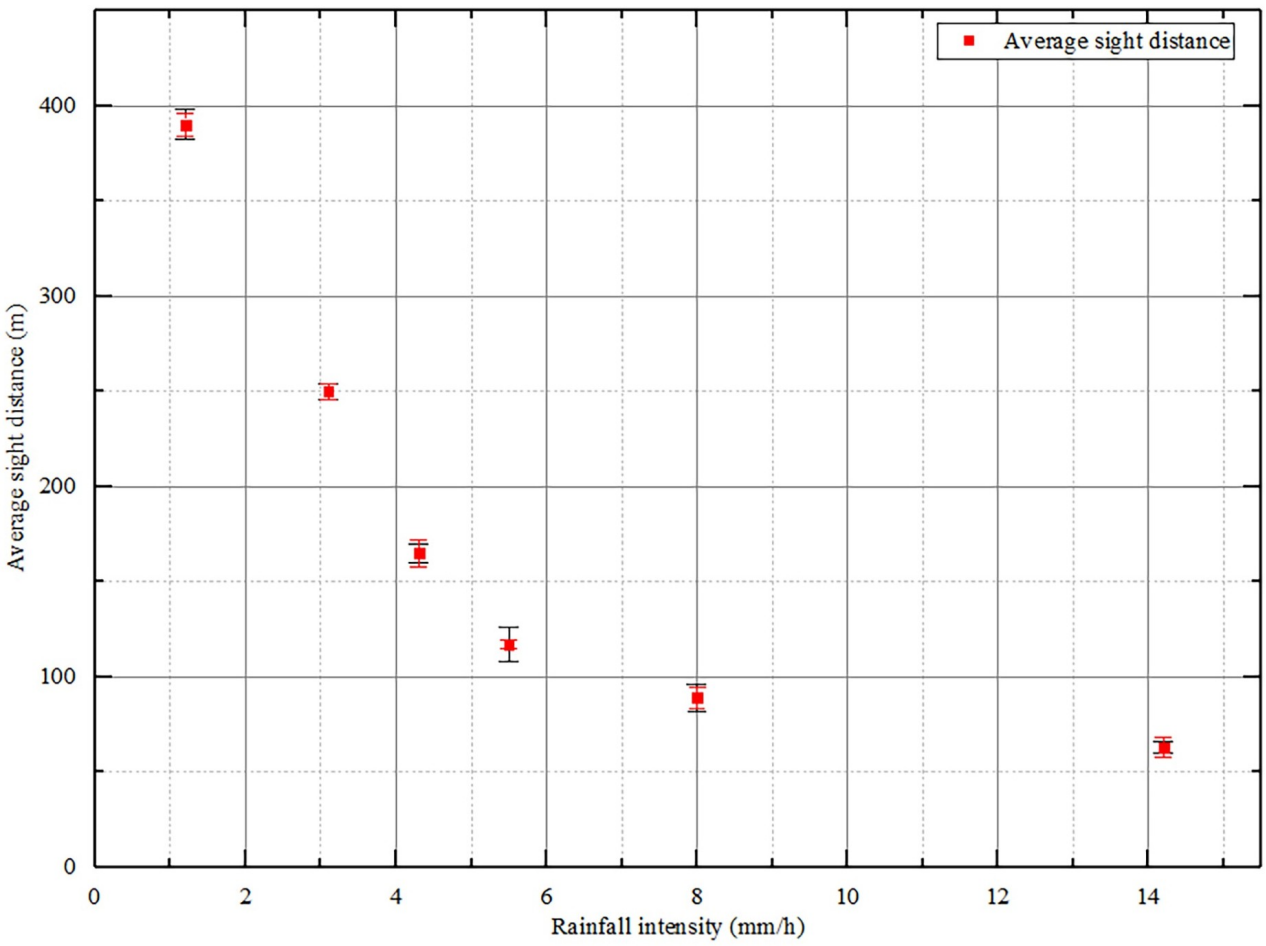
Average sight distance as a function of rainfall intensity.

Because the short-term heavy rainfall usually lasts one hour or even tens of minutes, only a total of 70 sets of sight distance data have been collected which is not enough for model fitting. Thus, this paper only conducts data regularity analysis without paying too much attention to the independent variables such as driver characteristics, geometric parameters, and vehicle characteristics (Fig 3).

When the visibility is shorter than road design sight distance caused by low visibility under adverse weather, the safety may be restricted with a high speed. China’s technical standard of highway engineering [46] stipulates that for expressways with a design speed of 120 km/h, the stopping sight distance on wet pavement is 210 meters. Researches show that when visibility is below 20 m, drivers are unable to complete normal driving operations [43]. Therefore, a sight distance range of 20–200 m is regarded as the research scope of this paper. Fig 3 indicates that when the rainfall intensity is less than 3.5mm/h, the sight distance is greater than the designed stopping sight distance of 210m. And the sight distance decreases rapidly with the increase of rainfall intensity. When the rainfall intensity is higher than 3.5mm/h, the sight distance is less than the demanded stopping sight distance. And the sight distance decreases slowly with the increase of rainfall intensity.

### 2.2 Lateral friction and rollover margins

Rollover and sideslips are two of the most common and severe forms of accidents related to vehicle lateral instability on horizontal curves. In general, whether a vehicle rollover or slips while negotiating curves is closely related to the height of the center of gravity and road friction, which is significantly affected by the weather. For all types of vehicles, the theoretical safe speed on combined alignments could be defined as the minimum value between the threshold sideslip speed and threshold rollover speed [21].

#### 2.2.1 Lateral friction margin

The lateral friction margin is defined as the difference between the available tire-pavement friction (i.e., side friction supply) and the friction demand (i.e., side friction factor) of the vehicle when negotiating curves.

$$FM=f_{\text{max}}-f$$ (4)

FM = Lateral friction margin

f_max_ = Maximum tire/pavement friction

f = Lateral friction factor

The side friction factor is the friction force divided by the component of the weight perpendicular to the pavement surface. It is expressed as a simplification of the basic curve formula considering the effect of super-elevation:

$$f=\frac{u^{2}}{127R}-i_{h}$$ (5)

R = Curve radius [m]

i_h_ = Superelevation [%/100]

This equation is based on the pavement-tire steering/cornering force analysis, which shows how the side friction factor acts as a counterbalance to the centripetal force developed as a vehicle performs a turning movement. The lateral friction factor has practical upper limits accommodating the safety and comfort of the intended users. Several studies aimed at determining the maximum side-friction factors that are comfortable for drivers have been conducted [47, 48]. But in the case of emergency braking, the upper limit of the side friction factor based on safety is the point of impending skid.

#### 2.2.2 Tire/ pavement friction under rainy weather

The pavement surface characteristics, tire properties, vehicle operational parameters, and the water film thickness are the main factors affecting the friction coefficient on wet roads. Among the four factors, only the water film thickness is a new factor compared with those on dry roads. The main source of water is rain. It can unfavorably remain under the tire as a hydrodynamic layer that separates the tire from the road, reducing the adhesion force and therefore, reducing friction. The relevant water film thickness is defined as the water layer height related to the road roughness peaks. Therefore, it can be negative if the water film is below the peaks or positive if it covers the peaks [49]. This paper focuses on the case where the water film thickness is positive under heavy short-term rainfall. The vehicle operational parameters combined with the water film thickness can completely determine the friction coefficient between tire and road in certain cases [49]. We adopted the equation determined by Hermann, S under block wheel state for the friction coefficient related to the vehicle speed and the water film thickness [50]:

$$f_{\text{max}}=0.241{\left(\frac{v}{100}\right)}^{2}-\left(0.721+0.297\text{lg}WFT\right)\left(\frac{v}{100}\right)+0.708+0.08\text{lg}WFT$$ (6)

v = Vehicle speed [m/s]

WFT = Water film thickness [mm]

Herrman defines the water film thickness as the film of water over the roughness top peak which corresponds to the positive relevant water film thickness as follows [50]:

WFT=h−MTD (7)

h = Water film height [mm]

MTD = Mean texture depth [mm]

The water film height is mainly influenced by the rainfall intensity but other factors should also be considered such as flow path length, road slope, and, texture [51, 52]. We choose to adopt Herman and Ressel Equation [50] with a variation of the inflow water, road slope, and types of the road with different textures to determine the water film height. The equation establishes a relationship between the water film height and the determined parameters based on a regression calculation established by conducting an experiment developed in a 2.5m long platform with 27 measure points:

$$h=0.263MTD^{0.4177}{\left(Lr_{i}\right)}^{0.4158}q^{-0.3314}$$ (8)

L = Flow path length [m]

r_i_ = Rainfall intensity [mm/h]

q = Road cross slope

China’s regulations on mean texture depth are as follows: the anti-skid mean texture depth of general sections of highway, first-class highway concrete pavement is not less than 0.7mm and not more than 1.1mm; the mean texture depth of asphalt concrete pavement is generally not less than 0.50mm.

The flow path length can be calculated as follows [52]:

$$L=b\frac{\sqrt{i^{2}+q^{2}}}{q}$$ (9)

b = Road width [m]

Any model able to predict the tire/ pavement friction under adverse weather (rain, fog, snow) could be used in the framework that we propose.

#### 2.2.3 Rollover margin

The rollover stability is characterized by rollover margin, which is defined based on lateral acceleration representing the difference between the current lateral acceleration and the maximum lateral acceleration that a vehicle could undergo without overturning is adopted to predict wheel lift for a vehicle traveling on a curve [48].

$$RM=a_{\text{max}}-a_{lat}$$ (10)

RM = Rollover margin

a_max_ = Upper limit of the lateral acceleration [m/s^2^]

a_lat_ = Lateral acceleration [m/s^2^]

While braking on straight sections allows the driver to mobilize all friction and energy in stopping, braking in curves requires mobilizing part of the friction to follow the path [53]. The vehicle turning on a horizontal curve undergoes a centripetal force that acts toward the center of curvature to keep the vehicle from sliding to the outside edge of the curve. This centripetal force is sustained by the tire/ pavement friction, by a component of the vehicle’s weight related to the roadway super-elevation, or by a combination of the two. This lateral acceleration can be calculated as the product of the side friction demand factor f_lat_ and the gravitational constant g [1] considering the effect of super-elevation based on the basic equation that governs vehicle operation on a curve from the laws of mechanics as follows:

$$a_{lat}=\frac{u^{2}}{3.6^{2}R}-i_{h}g$$ (11)

Lateral acceleration in gravitational units (g) is the static rollover threshold as the basic measure of roll stability. Although the rollover process of the vehicle is dynamic rather than quasi-static, the mass accident data and research show that the actual occurrence of rollover in accidents of heavy-truck is strongly related to the basic static roll stability of the vehicle [54, 55]. This lateral acceleration corresponds to the part of friction distributed to keep the trajectory in curves. Many researchers have studied the upper limit of the lateral acceleration to prevent rollover of the vehicle [11, 56]. Some of the results from these studies are listed in Table 1.

**Table 1:** Summary of the rollover threshold in existing researches.

Vehicle type		Rollover threshold
Passenger cars		1g
Light trucks, Vans, and SUVs		0.8~1.2g
Typical U.S. five-axle tractor-van semitrailer combination	Loaded to legal gross weight with a high-density, low center of gravity load	0.5g
	The worst-case load- one which fills the volume of the trailer while also reaching legal gross weight	0.25g
Typical U.S. five-axle petroleum semi-tanker		0.35g
Common cryogenic tankers for the transport of liquefied gases		0.26g
Logging trucks operating in Canada		0.23~0.31g

### 2.3 Combination of braking and turning maneuvers

The objective of this section is to comprehensively consider the longitudinal and lateral tire friction through the concept of friction circle or ellipse, so as to analyze the impact of the increase in longitudinal friction demand caused by the driver’s emergency braking operation on the lateral stability. The vector sum of the longitudinal and lateral force remains constant (circle) or near-constant (ellipse) referred to as the friction circle or friction ellipse [57]. As long as the vector sum of braking and turning friction components does not exceed the limits of tire grip as defined by the friction circle or friction ellipse, the amount of each component can vary independently. When operating at the limits of tire/pavement friction, the interaction of the longitudinal and lateral forces is that when one increases, the other must decrease by a proportional amount. Specifically, the application of longitudinal braking reduces the lateral force significantly, and vice versa [58]. As mentioned earlier, braking in curves requires mobilizing part of the friction to follow the path. The lateral friction is another important aspect of friction that occurs as the vehicle steers around a curve, changes lanes, or compensates for pavement cross-slope and/or crosswind effects. With combined braking and cornering maneuvers, vehicles with excessively high speeds may experience rear-end collisions or lateral instability. The relationship between the lateral friction, f_lat_, and the longitudinal friction, f_lon_, can be obtained as follows by resolving the road friction according to the vector:

$$f_{\text{max}}=\sqrt{f_{lat}^{2}+f_{lon}^{2}}$$ (12)

where

$$f_{lon}=\frac{a_{lon-\text{max}}}{g}$$ (13)

### 2.4 Determination of maximum safe vehicle speed

The objective of this section is to determine the permitted speed of single-unit trucks with emergency braking maneuver on horizontal curves under rainy weather, which is also the ultimate goal of this paper. This section explains how to determine the permitted speed based on the above longitudinal braking margin, lateral friction and rollover margins. As long as the margins, the visibility, and the friction under adverse weather are determined, the speed can be inversely calculated using measured local characteristics of the road (R, e, i), driver-related parameters (reaction time and driver’s pressure on the brake pedal), and vehicle-related parameters (the presence of an ABS). The safe speed in the case of emergency braking in curves under bad weather could be computed according to the rollover stability, the emergency braking capability, and the slip stability based on Eqs (2), (4) and (10) as conditions 1~3, respectively. On the one hand, the permitted speed should ensure that the vertical stopping distance is less than or equal to the sight distance, meanwhile, the vehicle does not roll or slip laterally. On the other hand, the distribution of vertical and horizontal friction should make the longitudinal and lateral maximum speeds as close as possible by adjusting the brake deceleration to maximize the utilization of friction.

Condition 1: Longitudinal braking margin

Make the longitudinal braking margin in Eq 2 equal to zero to obtain the permitted speed that meets the requirements of longitudinal braking distance.

$$\underset{a\prime }{\underset{︸}{\frac{1}{254\left(\frac{\gamma a_{lon-\text{max}}}{g}\pm i\right)}}}u_{1}^{2}+\underset{b\prime }{\underset{︸}{\frac{1}{3.6}\left(t_{1}+t_{2}^{\prime }+\frac{t_{2}^{\prime \prime }}{2}\right)}}u_{1}\underset{c\prime }{\underset{︸}{-s\prime }}=0$$ (14)

u_1_ = Permitted speed meeting the requirements of longitudinal braking distance [km/h]

a’ = The quadratic coefficient

b’ = The first-order coefficient

c’ = The constant term

It can be seen from Eq 14 that the sight distance requirement is a quadratic equation with one unknown about vehicle permitted speed. According to the root-finding formula of the quadratic equation in one variable, the permitted speed can be obtained as follows:

$$u_{1}=\frac{-b\prime +\sqrt{b\prime^{2}-4a\prime c\prime }}{2a\prime }$$ (15)

Noted that the permitted speed should be a positive value.

Condition 2: Lateral friction margin

It can be seen from Eq 6 that the friction coefficient is related to speed, so we substitute Eqs 5 and 6 into 4 to obtain the unary quartic equation about *u*_2_ as follows:

$$\underset{A}{\underset{︸}{\frac{1}{{\left(127R\right)}^{2}}}}u_{2}^{4}\underset{C}{\underset{︸}{-\left(\frac{2i_{h}}{127R}+\frac{0.241}{100^{2}}\right)}}u_{2}^{2}+\underset{D}{\underset{︸}{\frac{0.721+0.297\text{lg}WFT}{100}}}u_{2}+\underset{E}{\underset{︸}{i_{h}^{2}-0.708-0.081\text{lg}WFT+\frac{a_{lon}}{g}}}=0$$ (16)

u_2_ = Permitted speed meeting the requirements of lateral friction [km/h]

A = Coefficients of the quartic

C = Quadratic term

D = The first-order terms

E = A constant term

The root as the positive value of Eq 16 can be derived using Ferrari’s method is as follows:

Make

$$\{\begin{array}{l} \Delta_{1}=C^{2}+12AE\\ \Delta_{2}=2C^{3}+27AD^{2}-72ACE \end{array}$$ (17)

and

$$\Delta =\frac{\sqrt[{3}]{2}\Delta_{1}}{3A\sqrt[{3}]{\Delta_{2}+\sqrt{-4\Delta_{1}^{3}+\Delta_{2}^{2}}}}+\frac{\sqrt[{3}]{\Delta_{2}+\sqrt{-4\Delta_{1}^{3}+\Delta_{2}^{2}}}}{3\sqrt[{3}]{2}A}$$ (18)

therefore,

$$u_{2}=-\frac{1}{2}\sqrt{-\frac{2C}{3A}+\Delta }+\frac{1}{2}\sqrt{-\frac{4C}{3A}-\Delta +\frac{2D}{A\sqrt{-\frac{2C}{3A}+\Delta }}}$$ (19)

From the structure of the formula, the influencing factors are intricate and the physical meaning is difficult to determine. Permitted vehicle speed is related to road geometric parameters, water film thickness, braking deceleration, and other factors.

Condition 3: Rollover margin

Make the rollover margin in Eq 10 equal to zero to obtain the permitted speed that meets the requirements of rollover stability.

$$u_{3}=\sqrt{\left(a_{\text{max}}+gi_{h}\right)\times 12.96R}$$ (20)

u_3_ = Permitted speed meeting the requirements of rollover stability [km/h]

Therefore, the permitted speed of horizontal curves considering emergency braking maneuver under rainy weather is

$$u_{\text{max}}=\text{min}\left\{u_{1},u_{2},u_{3}\right\}$$ (21)

u_max_ = Permitted speed of horizontal curves considering emergency braking maneuver under rainy weather [km/h]

Model computation results were compared to simulation results to verify that the proposed mass-point model would correctly estimate the safe speed under the driver’s emergency braking maneuver in rainy weather.

## 3. Model validation

The simulation experiment can effectively avoid the safety risk of the driver driving in a really rainy environment, and it is a very economical and safer option compared to field studies, especially for vehicle operations that will not be safe to test in the real world. We adopted TruckSim multibody dynamics software to establish the dynamic simulation model. This software has been adopted by many international automobile manufacturers and component suppliers and has become the standard software in the automobile industry, enjoying a high reputation. The simulation consists of three essential components: the vehicle model, the 3D road model, and the driver control model. The detailed model establishment process and parameter settings will not be repeated here.

The single-unit truck is selected as the representative vehicle in this paper to validate the lateral stabilities in driving scenarios on curves because of its commonality in medium-distance missions with dangerous goods and the high risk of lateral instability. The test truck was an example of the TruckSim (Mechanical Simulation Corporation) vehicle data set, 4A LCF Van (SS-SS), Tandem F&R, 8×4. The values of related vehicle characteristics are the same as the model computation.

Road surfaces are perhaps the most critical part of the vehicle physics model providing the interface between the tires and ground. In the TruckSim math model, the concept of a road surface is mainly a representation of the ground properties: geometry and friction which are both dependent on the 3D road model. It should be noted that water film thickness and sight distance as input parameters of the calculation model cannot be directly simulated in TruckSim, but can be replaced by friction coefficient and braking distance respectively.

We chose “Procedure: Braking Distance Test” to simulate the driving behavior of a truck driver or an autonomous vehicle. First, the vehicle travels at a constant target speed equal to the permitted speed computed by the proposed model. Then, come to stop with constant deceleration obtained through trial calculation of the proposed model to ensure a balanced distribution of the friction coefficient in the longitudinal and lateral directions.

**Fig 4:**
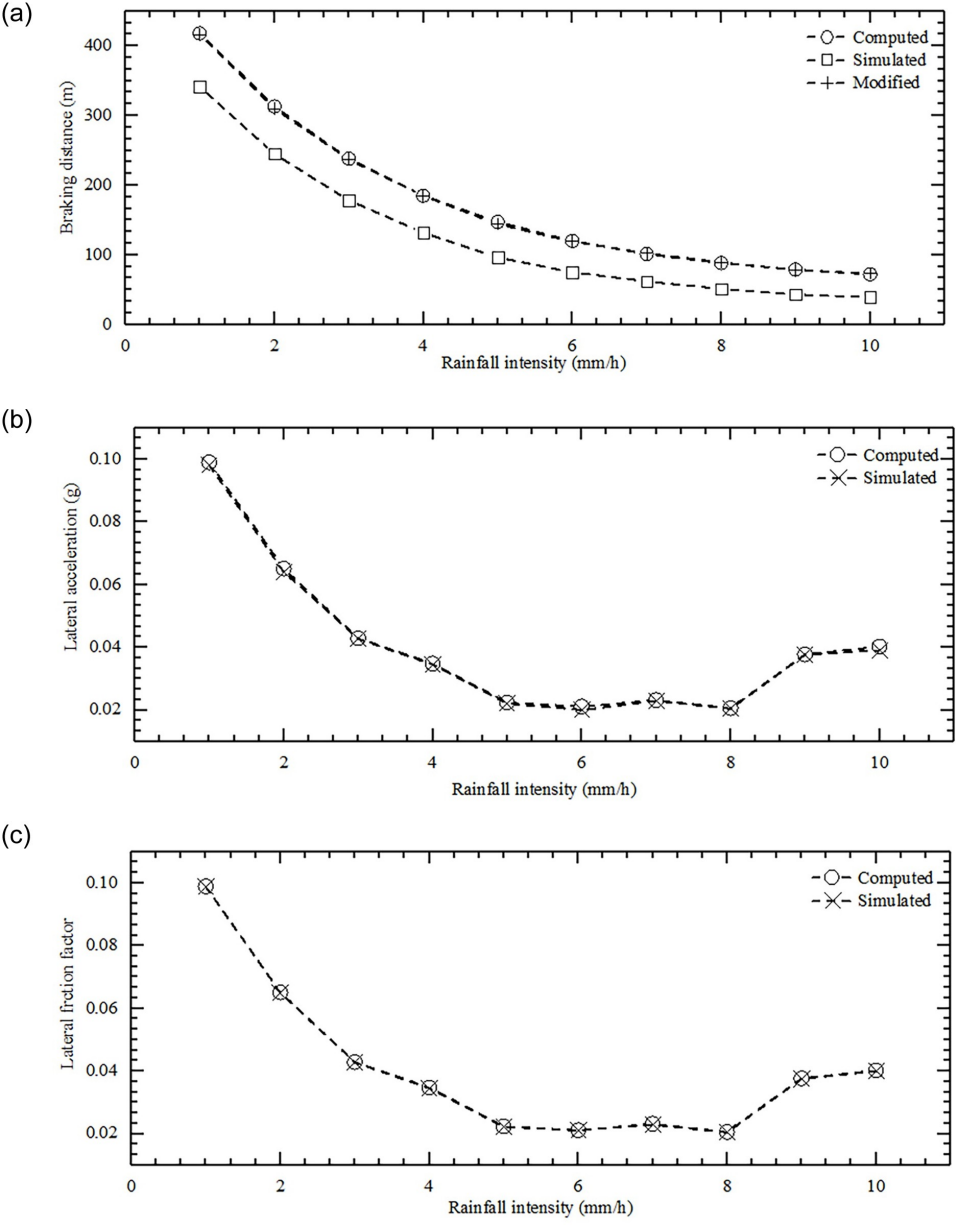
Comparison of the computed and simulated results. (a) Computed, modified, and simulated braking distance; (b) Computed and simulated lateral acceleration; (c) Computed and simulated lateral friction factor.

To ensure the validity of the verification, the input parameters and boundary conditions considered in the simulation test are as consistent as possible with the calculation model. The inputs of the simulation test are the brake deceleration, initial speed, and road friction data from the proposed model (see Table 2). The constant value of the environment, road, vehicle, and driver parameters remaining unchanged are listed in Table 3. The validity of the proposed model is verified by analyzing the simulation test results and model calculation values of the three critical conditions of braking distance, lateral acceleration, and tire lateral friction coefficient (Fig 4 and Table 4).

**Table 2:** The inputs of the simulation test.

Rainfall intensity (mm/h)	Road friction coefficient	Initial speed	Deceleration
		(km/h)	(m/s^2^)
1.0	0.214	109	1.87
2.0	0.210	94	1.96
3.0	0.225	85	2.17
4.0	0.248	77	2.41
5.0	0.273	70	2.67
6.0	0.298	64	2.92
7.0	0.312	58	3.06
8.0	0.330	64	3.24
9.0	0.347	51	3.39
10.0	0.361	49	3.52

**Table 3:** Parameter values.

	Parameter name	Symbol	Unit	Value
Driver-related parameters	The driver perception-reaction time	*τ*_1_	s	2.5
Vehicle-related parameters	The brake prepare time	$\tau_{2}^{\prime }$	s	0.3
	The brake applying time	$\tau_{2}^{\prime \prime }$	s	0.2
Environment-related parameters	Gravitational acceleration	*g*	m/s^2^	9.81
Road-related parameters	Superelevation	*i*_*h*_	%	8
	Curve radii	*R*	m	400
	Longitudinal slope	*i*	%	3
	Mean texture depth	MTD	mm	0.5
	Road width	b	m	15
The others	Driver and ABS-related parameter	*γ*	\	0.9

**Table 4:** Comparison of the computed and simulated results.

*r*_*i*_ (mm/h)	Computed values		Simulated values		Difference (%)		Magnitude of error	
	*S* (m)	*a*_*lat*_ (g)/ *f*_*lat*_	*S* (m)	*a*_*lat*_ (g)/ *f*_*lat*_	*δs* (m)	*δ a*_*lat*_ (g)	Δ*s* (m)	Δ *a*_*lat*_ (g)/ *f*_*lat*_
1.0	418	0.09896	416	0.098	0.0048	0.0098	2	0.00096
2.0	313	0.0651	310	0.064	0.0097	0.0172	3	0.0011
3.0	238	0.04284	237	0.0427	0.0042	0.0033	1	0.00014
4.0	185	0.03477	185	0.0345	0	0.0078	0	0.00027
5.0	147	0.02236	144	0.0221	0.0208	0.0118	3	0.00026
6.0	120	0.0211	119	0.02	0.0084	0.055	1	0.0011
7.0	101	0.02313	102	0.0229	-0.0098	0.0100	-1	0.00023
8.0	88	0.02057	89	0.02038	-0.0112	0.0093	-1	0.00019
9.0	79	0.03767	78	0.03764	0.0128	0.0008	1	3E-05
10.0	72	0.04022	73	0.039	-0.0137	0.0313	-1	0.00122

The graph of braking distance and rainfall intensity demonstrates that the simulated value is 34-75m lower than the computed value. By observing the simulation results of braking distance, it is found that the vehicle only experienced less than 0.5s from the moment when the vehicle started braking to that when the speed started to decrease. This is different from the consideration of 2.5-s driver’s perception-reaction time and 0.5-s brake reaction-prepare time in the process of establishing the proposed model. After adding 2.5-s of driving at a constant speed to the simulation results, the gap with the computed results will be reduced to 4m.

## 4. Conclusions

This paper has presented a procedure for the curve safe speed decision considering emergency braking maneuver under rainy weather, according to the vehicle longitudinal safety (restriction of the sight distance to the stopping distance) and the lateral safety (the balance of the road friction distribution in the vertical and horizontal directions). The results indicate that the effect of heavy rain on the permitted speed of the horizontal curve is mainly reflected in the decrease of the sight distance. In other words, the risk of rear-end collisions in rain is higher compared with rollover or sideslip accidents. Therefore, in the process of road design and construction, strengthening road drainage and reducing the decrease in sight distance is essential to driving safety on rainy days. The definition of “driver’s sight distance based on real-road scenarios” is safer than the visibility in the meteorological field. The established corresponding relationship between rainfall intensity-water film thickness-road friction makes it possible to determine the permitted speed directly based on the information issued by the weather station. This represents an important improvement over the AASHTO’s (2018) derivation of the effect of weather conditions: when the driver’s sight distance under adverse weather is lower than the demand stopping distance in AASHTO, the influence of emergency braking operation on the permitted speed below the design speed is considered. As well as those studies mentioned earlier focusing on the impacts of vehicle dynamics, road geometry, and environmental conditions on curve speed without considering the influence of driver behavior. It can be used to provide driving guidance for drivers on curves under rainy weather or as a decision-making basis for road managers. The shortcomings of this study are the insufficient collection of driver’s sight distance data due to weather restrictions. In addition, due to safety restrictions, it is difficult to carry out field tests for model verification.
